# Cytogenetic Study of Patients with Primary Amenorrhea in the Northeast of Iran

**DOI:** 10.30699/ijp.2020.115747.2258

**Published:** 2020-10-26

**Authors:** Narjes Soltani, Farzaneh Mirzaei, Hossein Ayatollahi

**Affiliations:** 1 *Cancer Molecular Pathology Research Center, Ghaem Medical Center Mashhad University of Medical Sciences, Mashhad, Iran*; 2 *Medical Genetic Research Center, School of Medicine, Mashhad University of Medical Sciences, Mashhad, Iran*

**Keywords:** Chromosomal abnormalities, Cytogenetic study, IRAN, Karyotyping, Primary amenorrhea

## Abstract

**Background & Objective:**

Primary amenorrhea refers to the absence of menstruation in females of reproductive by age 16 when the development of secondary sexual characteristics is evident (breast development, pubic hair) or by age 14 when there are no secondary sexual characteristics are present. Primary amenorrhea can occur in several quite different reasons. Common hormonal causes of primary amenorrhea include constitutional delay, hypothalamic or pituitary disorders, chronic systemic disease, and primary ovarian insufficiency, some endocrine gland disorders, and other causes. Previous studies suggested that chromosomal abnormality is the second most common cause of amenorrhea. This report aims to measure the prevalence of the chromosomal abnormality in primary amenorrhea (PA) patients in the northeast of Iran.

**Methods:**

Chromosomal study was carried out on 200 patients with clinical features. The standard method for culturing peripheral venous blood lymphocyte was to prepare metaphase chromosomes and perform routine GTG band analysis.

**Results:**

The results revealed that 71% of PA had normal female karyotype (46,XX) and 29% showed different chromosomal abnormalities. The chromosomal abnormalities can be categorized into seven primary groups with or without mosaicism. 1- The most common karyotype was X chromosome aneuploidy (10.5%, n=21), 2- Male karyotype with or without structural abnormality of Y chromosome (5.5 %, n=11), 3- Mosaicism of turner karyotype and structural anomalies of X chromosome (4%, n=8), 4- Structural anomalies of the X chromosome (3.5%, n=7), 5- Mosaicism of turner karyotype and normal karyotype (3%, n=6), 6- Mosaicism of turner karyotype and male karyotype (1.5%, n=3) and 7- Super female karyotype (1%, n=2).

**Conclusion:**

The present study has emphasized that early cytogenetic and timely investigation can be necessary for the evaluation of primary amenorrhea.

## Introduction

Primary amenorrhea is called non-menstruation at the age of 16 with normal growth of sexual characteristics or at the age of 14 with no growth or development of secondary sexual characteristics (1). 

There are many reasons for primary amenorrhea such as disorders of pituitary/hypothalamic (27.8%), gonadal dysfunction (50.4%), and outflow tract abnormalities (21.8%) (2). These abnormalities are caused by endocrine gland disorders such as thyroid or adrenal gland disorders and genetic, psychological, environmental, or structural anomalies. The cytogenetic investigation has shown that the frequency of chromosomal abnormalities varies from 15.9% to 63.3% in primary amenorrhea (3-6). The high diversity is probably due to various selection criteria in different reports. Today, with advances in cytogenetic techniques, the detection of chromosomal abnormalities is easier. Some cases with primary amenorrhea may have chromosomal abnormalities or patients are associated with sex reversal, i.e. patients with normal male chromosome complement but with female phenotype. The sex chromosome abnormalities may be numerical or structural, such as cases with abnormal small X chromosomes because of deletion or iso X chromosomes. There are some types of PA because of the mosaicism of the X chromosome such as X/XXX and X/XX (7, 8).

The object of this study, which was conducted in northeastern Iran, was to provide statistics and diagnosis of chromosomal abnormalities in patients with primary amenorrhea. Identifying these patients at an early age can be very helpful in accurately diagnosing and creating more successful treatment in these people.

##  Materials and Methods

**Sample Collection**

The study subjects including all patients with primary amenorrhea were referred to the Department of Molecular Pathology and Cytogenetics at Ghaem Hospital, Mashhad, Iran, from May 1^st^ 2011 to May 30^th^ 2019 were recruited. In addition, clinical information and laboratory research was provided from hospital records or patient physicians. The age group of the study participants ranged from 14 to 38 years with a mean of 20.34±5.86 years. At the initial visit, if the patient was diagnosed with PA, a physical examination was performed to identify secondary sexual characteristics or features of the syndrome. All patients expressed their voluntary informed consent according to the protocol approved by the Ethics Committee of Mashhad University of Medical Sciences (MUMS) to participate in the present study.

**Setting up of Lymphocyte Cultures**

Peripheral venous blood samples (in sterile conditions) were transferred into sterile culture tubes with 4 mL RPMI Medium 1640 with L-glutamine, (Gibco, Life Technologies, UK), 20% fetal bovine serum (Gibco, Life Technologies, UK), Penicillin–Streptomycin antibiotics solution, and Phytohaemagglutinin, stimulation of peripheral blood lymphocytes, (Gibco, Life Technologies, UK). For each sample, two culture tubes were placed, and then the cultures were incubated at 37 °. Forty-eight hours after culture initiation, blood cultures were synchronized with 100 microliters excess thymidine and returned to the incubator. After 16 hours, the thymidine is removed by rinsing. In the harvest stage, 4 hours later the cultured cells were incubated for 10 minutes after adding colcemid (Gibco, USA). Then suspensions of the cell were centrifuged at 1700 rpm for 10 min and immediately the pellets were incubated with a hypotonic solution (0.075 M KCl, Merck, Germany) for 15 minutes at 37ᵒC. The incubated cells were quickly fixed using Carnoy’s fixative solution (3:1 methanol-glacial acetic acid; Merck, Germany). The tubes again were centrifuged at 1700 rpm for 10 min and finally, the pellet was washed with Conroy’s fixative solution repeatedly 3 times (6).

**Slide Preparation and Karyotyping**

The slides prepared by the air-drop method and after 3 days of incubation, GTG banding was performed and the slides were colored with 10% Giemsa. 15 metaphase spreads were analyzed and examined (for each patient) with Video Test-Karyo software Version 3.1. In the analysis stage, 50 or 100 metaphases were examined when mosaicism was suspected. Karyotype results were reported based on the International System for Human Cytogenetic Nomenclature (ISCN) recommendations, 2016.

## Results

During this study, 200 female patients with PA were referred to the Department of Molecular Pathology and Cytogenetics at Ghaem Hospital for cytogenetic analysis. The age category referring to our center ranged from 14 to 38 years with a mean of 20.34 ± 5.86 years. The chromosomal analysis, karyotype, and frequency for the patients are shown in Table 1. The karyotype results demonstrated that 71% of patients have a normal chromosome structure (n=142) and 29% (n=58) have abnormal karyotype analysis. Table 2 compares the percentage of different karyotypes in patients with PA with the findings of other studies.

**Table 1:** Karyotype and frequency of the patients with PA

Cytogenetic variations	Karyotype	No. of cases	Frequency (%)
Normal karyotype	46,XX	142	71%
Monosomy X	45,X	21	10.5%
Turners mosaic	45,X/46,XX	6	3%
Super female karyotype	47,XXX	2	1%
Isochromosome X	46,X,i(X)(q10)	3	1.5%
Mosaicism of Turner and isochromosome X	45,X/46,X,i(X)(q10)	5	2.5%
Isodicentric X	46,X,idic(X)(q25)	2	1%
Mosaicism of turner and ring X	45,X/46,X,r(X)	3	1.5%
Sex reversal Sex reversal with structurally abnormal Y chromosome	46,XY 46,X,i(Y)(q10)	10 1	5% 0.5%
Mosaicism of turner and male karyotype	45,X/46,XY	3	1.5%
Deletion of X chromosome	46,X,del(X)(q12)	1	0.5%
X-autosome translocation	46,XX,t(X;18)(q24;p11.3)	1	0.5%

**Table 2:** Comparison of various chromosomal abnormalities found in patients with primary amenorrhea in different studies with present study

Author (year)	No .of case	X chromosome aneuploidy (%)	Structural anomalies (%)	Male Karyotype (%)	Abnormal chromosome (%)
**Roy and Banerjee (1995) (** 17 **)**	60	60	-	3.3	63.3
**Wong and Lam (2005) (** 18 **)**	237	12.7	2.9	8.4	24.5
**Rajangam and Nanjappa (2007) (** 5 **)**	865	45.54	12.27	31.19	23.35
**Safaei** *** et al.*** ** (2010) (** 6 **)**	220	10.9	3.6	6.4	20
**Kalavathi** *** et al.*** ** (2010) (** 19 **)**	979	10.41	6.9	6.23	23.39
**Vijayalakshmi** *** et al.*** ** (2010) (** 20 **)**	140	14.28	7.14	6.42	27.8
**Dutta** *** et al.*** ** (2013) (** 21 **)**	600	8.7	9.2	3	21
**Bhuyan** *** et al.*** ** (2012) (** 22 **)**	14	14.28	21.42	14.28	50
**Agacayak** *** et al.*** ** (2014) (** 23 **)**	108	2.5	20	-	22.5
**Malla** *** et al.*** ** (2016) (** 24 **)**	108	24.07	8.3	2.77	35.18

## Discussion

According to the World Health Organization, amenorrhea is the sixth prevalent cause of infertility in women, affecting about 2 to 5 percent of all women of childbearing age. Genetic abnormalities such as gene/chromosomal or multifactorial disorder are a major part of patients with primary amenorrhea. According to previous studies, the percentage of chromosomal abnormalities reported, varies greatly, from 15.9% to 63.3% for primary amenorrhea and this wide variation in the proportion is probably due to differences in the selection criteria for the patients (9). In rural areas of Iran, many patients with sexual dysfunction often do not seek medical advice and consider it as a social stigma, therefore the percentage and frequency of sexual abnormalities in Iranian patients with amenorrhea remain undetermined exactly. In the present study, we identified 29% chromosomal abnormalities among cases of PA, which are shown in accordance with the results obtained in several studies in Table 2.

In various published reports Turner syndrome is the leading cause of PA, either in form of pure 45,X or in its mosaic form. Turner syndrome is a disorder characterized by short stature, sexual infantilism, broad chests, a short and webbed neck, low hairline at the back of the neck, and low-set ears in adult women (25). Cell line 45,X formation is due to the meiotic non-disjunction or anaphase lagging of the reproductive cells in a parent or in early cell division during development (26). The deletion of the regions of chromosome X that are spared from X-inactivation (pseudoautosomal regions) make features of Turner syndrome (9). In this study, approximately 16% of the study population is detected to be Turner in mosaic or in pure form. The current study also reported two patients with primary amenorrhea with 47,XXX karyotype with no mental retardation, and other clinical or physical abnormalities. Non-mosaic 47,XXX cases were reported in premature ovarian failure cases in various studies (10). 

Among the reported structural abnormalities, 4% iso-chromosome of the long arm of X was found, which was in accordance with the results obtained in other studies shown in Table 2. Isochromosome formation is a relatively frequent chromosomal aberration, mainly in X chromosomes. The cause of this phenomenon is the division of the chromosomes transversely, not along their length. The resulting isochromosomes either have two short or two long arms so they have an unbalanced chromosomal constitution, monosomy for the missing, and trisomy for the duplicated arms (11). Isochromosomes may occur during mitosis and meiosis through a misdivision of the centromere or U-type strand exchange. Turner patients with the Isochromosome X constitution appear only in short stature and primary amenorrhea, so it really makes a challenge for physicians (27). Lack of normal phenotypic features of TS in these patients delays diagnosis and treatment with hormonal supplements. The ZFX gene on the short arm of the X chromosome is necessary for the normal development of female gonads (12).

Haploinsufficiency of the genes on the deleted regions of the X chromosome is the cause of gonadal dysgenesis. Spontaneous menarche did not appear in the patient with X long arm isochromosome, even in mosaicism with other cell lines (28). “The patient with i(Xq) have high plasma gonadotropin (FSH, LH) levels with low estradiol and progesterone level like classical turner syndrome (TS) patients. This hypertrophic hypogonadism phenomenon can be explained with the absence of feedback mechanism due to lack of ovarian functions” (13).

Male karyotype [46, XY] was detected in 11 cases (Swyer syndrome) in our study. Six of these XY female cases had normal female external genitalia, vagina, uterus, and fallopian tubes. In these cases, secondary sexual development did not occur at puberty, estrogens decreased and gonadotropins FSH and LH were elevated (14). On the Y chromosome, SRY is the testis determining factor (TDF) which causes sexual differentiation to the testis and its absence causes an inappropriate female gonad (29). Swyer syndrome is the exact reason for the unknown disorder in most cases. According to the studies, researchers have believed Swyer syndrome is caused by a mutation or disruption in gene or genes that are involved in normal sex differentiation of a fetus with an XY chromosomal constitution (30). The way of treatment is hormone therapy that is used in patients with primary gonadal failure. In women and girls with the syndrome Swyer, the most important concern is the risk of cancer in underdeveloped gonadal tissue. About 30% of females with this syndrome make a tumor that originates in the cells of ovaries or testis and gonadoblastoma is the most common gonadal tumor, so it is usually removed surgically early in life (15). Early diagnosis of this condition can help to timely hormonal treatment as well as prevent cancer. Successful fertilities are reported in 46,XY gender-reversed women (16).

Management of primary amenorrhea is depended on timely diagnosis and intervention based on the etiology. A complete investigation to recognize the reason for amenorrhea and timely hormonal and surgical treatment will make a better functional result. In patients with a chromosomal abnormality, detection of the kind of abnormality can help to provide the best treatment.

## Conclusion

It is clear that patients with these types of chromosomal abnormalities have a significant health, social and economic burden on themselves and their families. Thus cytogenetic diagnosis is becoming important for risk assessment and genetic counseling. Genetic counseling can be very helpful in guiding for the treatment or warning these patients about premature menopause for cases with Turner syndrome, the probability of infertility for patients with mosaic Turner, the prescription of hormone therapy, and the risk of gonadal malignancy for patients with XY dysgenesis. Initial examination and timely cytogenetic study can significantly contribute to life for patients, for instance initiating sex hormone replacement and growth hormone therapy for short stature patients with PA which may lead to a good answer. Furthermore, in cases of infertility and reproductive options, it has been reported in some cases that a successful response has been given to hormone therapy followed by fertility in PA patients with mosaic karyotype.
